# Supplementary material for: Challenges in the real world use of classification accuracy metrics: From recall and precision to the Matthews correlation coefficient
Source: PLoS One. 2023 Oct 4;18(10):e0291908. doi: 10.1371/journal.pone.0291908 (PMC10550141; doi:10.1371/journal.pone.0291908)
Supplement: S1 Table — (DOCX) [file pone.0291908.s001.docx]

| Data to form the confusion matrices for the scenarios using independent error.  Black - outcome if gold standard had been used  Blue - imperfect reference (accuracy =0.98) used  Green - imperfect reference (accuracy =0.90) used  Red - imperfect reference (accuracy =0.82) used   \|  \| \| --- \| |  |  |  |  |  |  |  |  |  |  |  |  |  |  |  |  |
| --- | --- | --- | --- | --- | --- | --- | --- | --- | --- | --- | --- | --- | --- | --- | --- | --- | --- |
|  |  |  |  |  |  |  |  |  |  |  |  |  |  |  |  |  |
|  |  |  |  |  |  |  |  |  |  |  |  |  |  |  |  |  |
|  |  |  |  |  |  |  |  |  |  |  |  |  |  |  |  |  |
|  |  |  |  |  |  |  |  |  |  |  |  |  |  |  |  |  |
|  |  |  |  |  |  |  |  |  |  |  |  |  |  |  |  |  |
|  |  |  |  |  |  |  |  |  |  |  |  |  |  |  |  |  |
|  |  |  |  |  |  |  |  |  |  |  |  |  |  |  |  |  |
|  |  |  |  |  |  |  |  |  |  |  |  |  |  |  |  |  |
| Prevalence | TP | FP | FN | TN | TP | FP | FN | TN | TP | FP | FN | TN | TP | FP | FN | TN |
| 0.01 | 8 | 198 | 2 | 792 | 11.8 | 194.2 | 17.8 | 776 | 27 | 179 | 81 | 713 | 42.2 | 163.8 | 144.2 | 649.8 |
| 0.05 | 40 | 190 | 10 | 760 | 43 | 187 | 25 | 745 | 55 | 175 | 85 | 685 | 67 | 163 | 145 | 625 |
| 0.1 | 80 | 180 | 20 | 720 | 82 | 178 | 34 | 706 | 90 | 170 | 90 | 650 | 98 | 162 | 146 | 594 |
| 0.15 | 120 | 170 | 30 | 680 | 121 | 169 | 43 | 667 | 125 | 165 | 95 | 615 | 129 | 161 | 147 | 563 |
| 0.2 | 160 | 160 | 40 | 640 | 160 | 160 | 52 | 628 | 160 | 160 | 100 | 580 | 160 | 160 | 148 | 532 |
| 0.25 | 200 | 150 | 50 | 600 | 199 | 151 | 61 | 589 | 195 | 155 | 105 | 545 | 191 | 159 | 149 | 501 |
| 0.3 | 240 | 140 | 60 | 560 | 238 | 142 | 70 | 550 | 230 | 150 | 110 | 510 | 222 | 158 | 150 | 470 |
| 0.35 | 280 | 130 | 70 | 520 | 277 | 133 | 79 | 511 | 265 | 145 | 115 | 475 | 253 | 157 | 151 | 439 |
| 0.4 | 320 | 120 | 80 | 480 | 316 | 124 | 88 | 472 | 300 | 140 | 120 | 440 | 284 | 156 | 152 | 408 |
| 0.45 | 360 | 110 | 90 | 440 | 355 | 115 | 97 | 433 | 335 | 135 | 125 | 405 | 315 | 155 | 153 | 377 |
| 0.5 | 400 | 100 | 100 | 400 | 394 | 106 | 106 | 394 | 370 | 130 | 130 | 370 | 346 | 154 | 154 | 346 |
| 0.55 | 440 | 90 | 110 | 360 | 433 | 97 | 115 | 355 | 405 | 125 | 135 | 335 | 377 | 153 | 155 | 315 |
| 0.6 | 480 | 80 | 120 | 320 | 472 | 88 | 124 | 316 | 440 | 120 | 140 | 300 | 408 | 152 | 156 | 284 |
| 0.65 | 520 | 70 | 130 | 280 | 511 | 79 | 133 | 277 | 475 | 115 | 145 | 265 | 439 | 151 | 157 | 253 |
| 0.7 | 560 | 60 | 140 | 240 | 550 | 70 | 142 | 238 | 510 | 110 | 150 | 230 | 470 | 150 | 158 | 222 |
| 0.75 | 600 | 50 | 150 | 200 | 589 | 61 | 151 | 199 | 545 | 105 | 155 | 195 | 501 | 149 | 159 | 191 |
| 0.8 | 640 | 40 | 160 | 160 | 628 | 52 | 160 | 160 | 580 | 100 | 160 | 160 | 532 | 148 | 160 | 160 |
| 0.85 | 680 | 30 | 170 | 120 | 667 | 43 | 169 | 121 | 615 | 95 | 165 | 125 | 563 | 147 | 161 | 129 |
| 0.9 | 720 | 20 | 180 | 80 | 706 | 34 | 178 | 82 | 650 | 90 | 170 | 90 | 594 | 146 | 162 | 98 |
| 0.95 | 760 | 10 | 190 | 40 | 745 | 25 | 187 | 43 | 685 | 85 | 175 | 55 | 625 | 145 | 163 | 67 |
| 0.99 | 792 | 2 | 198 | 8 | 776 | 17.8 | 194.2 | 11.8 | 713 | 81 | 179 | 27 | 650 | 144.2 | 163.8 | 42.2 |
