# Supplementary material for: Challenges in the real world use of classification accuracy metrics: From recall and precision to the Matthews correlation coefficient
Source: PLoS One. 2023 Oct 4;18(10):e0291908. doi: 10.1371/journal.pone.0291908 (PMC10550141; doi:10.1371/journal.pone.0291908)
Supplement: S2 Table — (DOCX) [file pone.0291908.s002.docx]

| Data to form the confusion matrices for the scenarios using correlated error.  Black - outcome if gold standard had been used  Blue - imperfect reference (accuracy =0.98) used  Green - imperfect reference (accuracy =0.90) used  Red - imperfect reference (accuracy =0.82) used   \|  \| \| --- \| |  |  |  |  |  |  |  |  |  |  |  |  |  |  |  |  |
| --- | --- | --- | --- | --- | --- | --- | --- | --- | --- | --- | --- | --- | --- | --- | --- | --- | --- |
|  |  |  |  |  |  |  |  |  |  |  |  |  |  |  |  |  |
|  |  |  |  |  |  |  |  |  |  |  |  |  |  |  |  |  |
|  |  |  |  |  |  |  |  |  |  |  |  |  |  |  |  |  |
|  |  |  |  |  |  |  |  |  |  |  |  |  |  |  |  |  |
|  |  |  |  |  |  |  |  |  |  |  |  |  |  |  |  |  |
|  |  |  |  |  |  |  |  |  |  |  |  |  |  |  |  |  |
|  |  |  |  |  |  |  |  |  |  |  |  |  |  |  |  |  |
|  |  |  |  |  |  |  |  |  |  |  |  |  |  |  |  |  |
| Prevalence | TP | FP | FN | TN | TP | FP | FN | TN | TP | FP | FN | TN | TP | FP | FN | TN |
| 0.01 | 8 | 198 | 2 | 792 | 27.8 | 178 | 1.8 | 792 | 107 | 99 | 1 | 793 | 186 | 19.8 | 0.2 | 794 |
| 0.05 | 40 | 190 | 10 | 760 | 59 | 171 | 9 | 761 | 135 | 95 | 5 | 765 | 211 | 19 | 1 | 769 |
| 0.1 | 80 | 180 | 20 | 720 | 98 | 162 | 18 | 722 | 170 | 90 | 10 | 730 | 242 | 18 | 2 | 738 |
| 0.15 | 120 | 170 | 30 | 680 | 137 | 153 | 27 | 683 | 205 | 85 | 15 | 695 | 273 | 17 | 3 | 707 |
| 0.2 | 160 | 160 | 40 | 640 | 176 | 144 | 36 | 644 | 240 | 80 | 20 | 660 | 304 | 16 | 4 | 676 |
| 0.25 | 200 | 150 | 50 | 600 | 215 | 135 | 45 | 605 | 275 | 75 | 25 | 625 | 335 | 15 | 5 | 645 |
| 0.3 | 240 | 140 | 60 | 560 | 254 | 126 | 54 | 566 | 310 | 70 | 30 | 590 | 366 | 14 | 6 | 614 |
| 0.35 | 280 | 130 | 70 | 520 | 293 | 117 | 63 | 527 | 345 | 65 | 35 | 555 | 397 | 13 | 7 | 583 |
| 0.4 | 320 | 120 | 80 | 480 | 332 | 108 | 72 | 488 | 380 | 60 | 40 | 520 | 428 | 12 | 8 | 552 |
| 0.45 | 360 | 110 | 90 | 440 | 371 | 99 | 81 | 449 | 415 | 55 | 45 | 485 | 459 | 11 | 9 | 521 |
| 0.5 | 400 | 100 | 100 | 400 | 410 | 90 | 90 | 410 | 450 | 50 | 50 | 450 | 490 | 10 | 10 | 490 |
| 0.55 | 440 | 90 | 110 | 360 | 449 | 81 | 99 | 371 | 485 | 45 | 55 | 415 | 521 | 9 | 11 | 459 |
| 0.6 | 480 | 80 | 120 | 320 | 488 | 72 | 108 | 332 | 520 | 40 | 60 | 380 | 552 | 8 | 12 | 428 |
| 0.65 | 520 | 70 | 130 | 280 | 527 | 63 | 117 | 293 | 555 | 35 | 65 | 345 | 583 | 7 | 13 | 397 |
| 0.7 | 560 | 60 | 140 | 240 | 566 | 54 | 126 | 254 | 590 | 30 | 70 | 310 | 614 | 6 | 14 | 366 |
| 0.75 | 600 | 50 | 150 | 200 | 605 | 45 | 135 | 215 | 625 | 25 | 75 | 275 | 645 | 5 | 15 | 335 |
| 0.8 | 640 | 40 | 160 | 160 | 644 | 36 | 144 | 176 | 660 | 20 | 80 | 240 | 676 | 4 | 16 | 304 |
| 0.85 | 680 | 30 | 170 | 120 | 683 | 27 | 153 | 137 | 695 | 15 | 85 | 205 | 707 | 3 | 17 | 273 |
| 0.9 | 720 | 20 | 180 | 80 | 722 | 18 | 162 | 98 | 730 | 10 | 90 | 170 | 738 | 2 | 18 | 242 |
| 0.95 | 760 | 10 | 190 | 40 | 761 | 9 | 171 | 59 | 765 | 5 | 95 | 135 | 769 | 1 | 19 | 211 |
| 0.99 | 792 | 2 | 198 | 8 | 792 | 1.8 | 178 | 27.8 | 793 | 1 | 99 | 107 | 794 | 0.2 | 19.8 | 186 |
