# Supplementary material for: Challenges in the real world use of classification accuracy metrics: From recall and precision to the Matthews correlation coefficient
Source: PLoS One. 2023 Oct 4;18(10):e0291908. doi: 10.1371/journal.pone.0291908 (PMC10550141; doi:10.1371/journal.pone.0291908)
Supplement: S3 Table — (DOCX) [file pone.0291908.s003.docx]

| Data to form the confusion matrices for the assessment of the poor classification (Fig 7).  Black - outcome if gold standard had been used  Orange - imperfect reference (accuracy =0.7) used   \|  \| \| --- \| |  |  |  |  |  |  |  |  |
| --- | --- | --- | --- | --- | --- | --- | --- | --- | --- |
|  |  |  |  |  |  |  |  |  |
|  |  |  |  |  |  |  |  |  |
|  |  |  |  |  |  |  |  |  |
|  |  |  |  |  |  |  |  |  |
|  |  |  |  |  |  |  |  |  |
| Prevalence | TP | FP | FN | TN | TP | FP | FN | TN |
| 0.01 | 5 | 495 | 5 | 495 | 302 | 198 | 2 | 498 |
| 0.05 | 25 | 475 | 25 | 475 | 310 | 190 | 10 | 490 |
| 0.1 | 50 | 450 | 50 | 450 | 320 | 180 | 20 | 480 |
| 0.15 | 75 | 425 | 75 | 425 | 330 | 170 | 30 | 470 |
| 0.2 | 100 | 400 | 100 | 400 | 340 | 160 | 40 | 460 |
| 0.25 | 125 | 375 | 125 | 375 | 350 | 150 | 50 | 450 |
| 0.3 | 150 | 350 | 150 | 350 | 360 | 140 | 60 | 440 |
| 0.35 | 175 | 325 | 175 | 325 | 370 | 130 | 70 | 430 |
| 0.4 | 200 | 300 | 200 | 300 | 380 | 120 | 80 | 420 |
| 0.45 | 225 | 275 | 225 | 275 | 390 | 110 | 90 | 410 |
| 0.5 | 250 | 250 | 250 | 250 | 400 | 100 | 100 | 400 |
| 0.55 | 275 | 225 | 275 | 225 | 410 | 90 | 110 | 390 |
| 0.6 | 300 | 200 | 300 | 200 | 420 | 80 | 120 | 380 |
| 0.65 | 325 | 175 | 325 | 175 | 430 | 70 | 130 | 370 |
| 0.7 | 350 | 150 | 350 | 150 | 440 | 60 | 140 | 360 |
| 0.75 | 375 | 125 | 375 | 125 | 450 | 50 | 150 | 350 |
| 0.8 | 400 | 100 | 400 | 100 | 460 | 40 | 160 | 340 |
| 0.85 | 425 | 75 | 425 | 75 | 470 | 30 | 170 | 330 |
| 0.9 | 450 | 50 | 450 | 50 | 480 | 20 | 180 | 320 |
| 0.95 | 475 | 25 | 475 | 25 | 490 | 10 | 190 | 310 |
| 0.99 | 495 | 5 | 495 | 5 | 498 | 2 | 198 | 302 |
