# Supplementary material for: Challenges in the real world use of classification accuracy metrics: From recall and precision to the Matthews correlation coefficient
Source: PLoS One. 2023 Oct 4;18(10):e0291908. doi: 10.1371/journal.pone.0291908 (PMC10550141; doi:10.1371/journal.pone.0291908)
Supplement: Attachment — Submitted filename: PONE Review.docx [file pone.0291908.s004.docx]

Manuscript Number: PONE-D-23-13169

Reviewer’s Concern # 1: First of all, include the introductory sentence about the problem statement in abstract.

Reviewer’s Concern # 2: In the second part, the author should give an overall description of the entire technique first, then give the point-by-point introduction to these used techniques.

Reviewer’s Concern # 3: The author should define all performance measures first, then compare the results of different schemes.

Reviewer’s Concern # 4: What is the research significance of this article?

Reviewer’s Concern # 5: There is little literature in the past three years, and the latest research should be cited.

Reviewer’s Concern # 6: Correct the references and follow the reference format of PLOS ONE.

Reviewer’s Concern # 7: The authors should include a more extensive discussion of the novelty of their system in Introduction Part.

Reviewer’s Concern # 8: The English language needs to be seriously improved. Some sentences are incomplete, others have different meaning than the authors intended to. Also, the authors should try to shorten some of the sentences. Many of them are too long and are not easy to follow and understand.

Reviewer’s Concern # 9: The structure of the paper is, in my opinion, too confusing, too complex. The introduction should be shorter and much and more to the point. It fails to do the one of the most important roles of the introduction, it fails to present sufficient information, so that important readers and evaluators do not need to read the rest of the text, being assured in the contribution made, and validity of the text to follow. It should establish the paper’s motivation, methods, contribution, results, and position in scientific literature. For each of the sections, similar comments could be made.

Reviewer’s Concern # 10: Include Discussion section, which is missing.

Reviewer’s Concern # 11: The Conclusion is very lengthy. Briefly elaborate it in a paragraph.
