# Supplementary material for: Challenges in the real world use of classification accuracy metrics: From recall and precision to the Matthews correlation coefficient
Source: PLoS One. 2023 Oct 4;18(10):e0291908. doi: 10.1371/journal.pone.0291908 (PMC10550141; doi:10.1371/journal.pone.0291908)
Supplement: Attachment — Submitted filename: Response to reviewers.docx [file pone.0291908.s005.docx]

**Response to reviewer’s comments**

Thank you for the comments on the article. These comments have informed the revision of the article and below a brief response is provided to each point. For completeness and clarity, the full set of comments received is copied below and a brief response made on an essentially point-by-point basis. The responses are provided in *red italics* to help separate them from the comments.

**Comments to the Author**

1. Is the manuscript technically sound, and do the data support the conclusions?

The manuscript must describe a technically sound piece of scientific research with data that supports the conclusions. Experiments must have been conducted rigorously, with appropriate controls, replication, and sample sizes. The conclusions must be drawn appropriately based on the data presented.

Reviewer #1: Partly

Reviewer #2: Partly

*The revisions made to the specific comments made by the reviewers that are reported below hopefully address their concerns.*

2. Has the statistical analysis been performed appropriately and rigorously?

Reviewer #1: No

Reviewer #2: No

*This paper does not include any statistical testing.*

3. Have the authors made all data underlying the findings in their manuscript fully available?

The [PLOS Data policy](http://www.plosone.org/static/policies.action#sharing) requires authors to make all data underlying the findings described in their manuscript fully available without restriction, with rare exception (please refer to the Data Availability Statement in the manuscript PDF file). The data should be provided as part of the manuscript or its supporting information, or deposited to a public repository. For example, in addition to summary statistics, the data points behind means, medians and variance measures should be available. If there are restrictions on publicly sharing data—e.g. participant privacy or use of data from a third party—those must be specified.

Reviewer #1: No

Reviewer #2: No

*The revisions made to the specific comments made by the reviewers hopefully address their concerns. Please note that the article uses a simulation-based approach and hence there is no data. The methods used and equations provided allow the work to be fully replicated if desired.*

4. Is the manuscript presented in an intelligible fashion and written in standard English?

PLOS ONE does not copyedit accepted manuscripts, so the language in submitted articles must be clear, correct, and unambiguous. Any typographical or grammatical errors should be corrected at revision, so please note any specific errors here.

Reviewer #1: Yes

Reviewer #2: Yes

*Thank you. Some revsion to further enhance the text has been undertaken as part of the response to the review comments recieved.*

5. Review Comments to the Author

Please use the space provided to explain your answers to the questions above. You may also include additional comments for the author, including concerns about dual publication, research ethics, or publication ethics. (Please upload your review as an attachment if it exceeds 20,000 characters)

Reviewer #1: Reviewer’s Concern # 1: First of all, include the introductory sentence about the problem statement in abstract.
Reviewer’s Concern # 2: In the second part, the author should give an overall description of the entire technique first, then give the point-by-point introduction to these used techniques.
Reviewer’s Concern # 3: The author should define all performance measures first, then compare the results of different schemes.
Reviewer’s Concern # 4: What is the research significance of this article?

*Thank you for these comments. The core thrust of concerns #1 to #4 seem to relate to the opening text. The length limitation on the abstract (300 words) makes it difficult to accommodate new material and issues such as the vast range of different terminology used in the literature make it challenging to define performance measures any earlier. However, to try and address the concerns flagged in #1 to #4 plus #7 and #9 the opening part of the Introduction has been completely changed. A full page of new text has been added that aims to state the problem, indicate briefly key contributions and significance.*

Reviewer’s Concern # 5: There is little literature in the past three years, and the latest research should be cited.

*The article is explicit that many of the issues are well known and does not seek to claim novelty on the fundamental issues. Reference is deliberately made to old articles as these are the core references that underpin the work and also act to highlight the inaction of the research community to known problems. Note also that in the original submission over 30% of the articles cited were published 2020-2023.*

Reviewer’s Concern # 6: Correct the references and follow the reference format of PLOS ONE.

*Thank you for the comment. Some references were written in an incorrect style. These references are now written in the correct format.*

Reviewer’s Concern # 7: The authors should include a more extensive discussion of the novelty of their system in Introduction Part.

*The new opening text addresses this concern. As noted above the article is based on well-known issues and is not claiming novelty – it is seeking to help raise awareness and encourage good practice.*

Reviewer’s Concern # 8: The English language needs to be seriously improved. Some sentences are incomplete, others have different meaning than the authors intended to. Also, the authors should try to shorten some of the sentences. Many of them are too long and are not easy to follow and understand.

*Thank you for the comment. The text has been revised with some sentences shortened.*

Reviewer’s Concern # 9: The structure of the paper is, in my opinion, too confusing, too complex. The introduction should be shorter and much and more to the point. It fails to do the one of the most important roles of the introduction, it fails to present sufficient information, so that important readers and evaluators do not need to read the rest of the text, being assured in the contribution made, and validity of the text to follow. It should establish the paper’s motivation, methods, contribution, results, and position in scientific literature. For each of the sections, similar comments could be made.

*The new opening text addresses this concern.*

Reviewer’s Concern # 10: Include Discussion section, which is missing.
Reviewer’s Concern # 11: The Conclusion is very lengthy. Briefly elaborate it in a paragraph.

*Concerns #10 and #11 focus on the final sections of the article. It may be possible that the referee missed the ‘Discussion’ heading in the original submission and viewed the Discussion and Conclusion sections as one? The revised manuscript has been checked to ensure that the section headings are present and clear.*

*I am grateful to the referee for the comments and hope that the concerns raised have been addressed.*

Reviewer #2: While the topic addressed in this paper is certainly important, I feel the treatment of the details was somewhat underwhelming. Specifically, I would urge the author to add the following to the manuscript:

1.Reading through the paper, there were minimal and negligible insights into why the different parameters behave the way they do. For instance, sensitivity and specificity are theoretically defined within two subsets of the population - positives and negatives based on the reference standards. In that case, these parameters should be theoretically independent of prevalence. Yet, in practice we do know (as the author also notes) that sensitivity and specificity are affected or influenced by prevalence. A more detailed description of why such behavior is seen is what I was really looking forward to.

*Thank you for the comment. The issue flagged is a really important one – sensitivity (or Recall) and specificity are widely quoted as being independent of prevalence. Indeed, these metrics are theoretically independent of prevalence and this can also be observed BUT only if a true gold standard reference is used. It is the use of an imperfect reference that causes prevalence dependency. To try to explicitly address this issue four new paragraphs have been added early to the Discussion section. These paragraphs seek to explain the effects of the imperfect reference on the manner in which the cases are distributed in the confusion matrix and how this impacts on the magnitude of the selected metrics. The discussion expands the discussion of Fig 2 which is designed to summarise key issues with example confusion matrices.*

2. In clinical epidemiology, the concept of positive and negative likelihood ratios is fairly well established. The LRs provide a clinically meaningful interpretation that is minimally influenced by prevalence. I did not find any discussion or reference to the LRs in the paper. I suggest that the author add LRs to the same data used in the paper and demonstrate the relative robustness of these parameters in classification tasks.

*There are many additional metrics of accuracy that could be considered. Many like LRs are based on sensitivity and specificity and so will be impacted by the use of an imperfect reference. LRs have now been included as an example. Key changes made include the addition of LRs to the section 1.2 in which the set of metrics investigated are defined and Fig 8 that shows the results for the LRs using the same set of simulated imperfect reference standards.*

3. I feel the paper does not end with specific recommendations for authors. That the use of these parameters is fraught with dangers is well known. What a researcher actually needs to do when confronted with the situation is not clear from the paper. The value of the paper will be greatly improved if such recommendations - based on real or simulated data - can be categorically spelt out.

*The Conclusion section has been revised to enhance the discussion on what researchers should do and also expanded to encourage readers to be cautious of published results. The new opening text added to address issues flagged by the other referee also stresses what researchers should do. Note also the text does highlight key issues such as the desire to correct estimates and points to several articles that provide a fuller discussion. Specific examples include lines 63-67, 143-145, 176-178, 701-714 and 768-769 which point the reader to a variety of articles that discuss the issue notably references 2, 16, 17, 20, 29, 30, 31, 32, 45, 62, 63, and 64.*

*I am grateful to the referee for the comments and hope that the concerns raised have been addressed.*

6. PLOS authors have the option to publish the peer review history of their article ([what does this mean?](https://journals.plos.org/plosone/s/editorial-and-peer-review-process#loc-peer-review-history)). If published, this will include your full peer review and any attached files.


If you choose “no”, your identity will remain anonymous but your review may still be made public.


**Do you want your identity to be public for this peer review?** For information about this choice, including consent withdrawal, please see our [Privacy Policy](https://www.plos.org/privacy-policy).

Reviewer #1: No

Reviewer #2: No

*I am grateful to the referees for their helpful and also quick review comments.*
