# Supplementary material for: Challenges in the real world use of classification accuracy metrics: From recall and precision to the Matthews correlation coefficient
Source: PLoS One. 2023 Oct 4;18(10):e0291908. doi: 10.1371/journal.pone.0291908 (PMC10550141; doi:10.1371/journal.pone.0291908)
Supplement: Attachment — Submitted filename: Response to reviewers on revised manuscript.docx [file pone.0291908.s006.docx]

**Response to reviewer’s comments**

Thank you for the comments on the revised article. These comments have informed the further revision of the article and below a brief response is provided to each point. For completeness and clarity, the full set of comments received is copied below and a brief response made on an essentially point-by-point basis. The responses are provided in *red italics* to help separate them from the comments.

**Comments to the Author**

6. Review Comments to the Author

Please use the space provided to explain your answers to the questions above. You may also include additional comments for the author, including concerns about dual publication, research ethics, or publication ethics. (Please upload your review as an attachment if it exceeds 20,000 characters)

Reviewer #1: Some minor edits required in spellings and grammar mistakes and also correct the references style according to PLOS ONE journal. You may cite latest articles in your manuscript, e.g.
1. <https://doi.org/10.1371/journal.pone.0240015>
2. <https://doi.org/10.3390/s22072724>
3. <https://doi.org/10.1109/ACCESS.2021.3084905>

*Thank you for the comments. Some minor editing has been undertaken. The suggested references are interesting and use some of the metrics discussed in the article but their inclusion would not add constructively; the suggested articles have not been cited.*

Reviewer #3: While I was not a reviewer for the first version of the manuscript, I was invited to review Revision 1. I believe the author has adequately addressed all comments from the original reviewers. My recommendation of "Minor Revision" arises from a sentence in Rev1. Lines 763-765 state that this article has shown that a high MCC value does not necessarily imply good classification performance in both classes of a binary classification problem. However, lines 689-691 explicitly mention that this is to be expected in the case of a gold standard reference. Hence, the conclusions should be changed to indicate that MCC will have poor performance with inaccurate datasets and grossly imbalanced classes.

*Thank you for the comment. I agree that highlighting the use of an imperfect reference is critical here and hence the text in the conclusions has been revised. The relevant paragraph in the (clean) version of the manuscript on lines 760-768 now reads:*

*“Of particular importance, however, is that the MCC displayed undesirable properties. It was possible for the apparent value of the MCC to be substantially under- or over-estimated as a result of variations in prevalence and/or use of an imperfect reference. Critically, a high value for the MCC could be obtained from a poor classification, notably* ***if the reference standard used was inaccurate with errors correlated with those in the classification under evaluation****. This observation runs contrary to arguments promoting the MCC that contend that a high value is only possible when the classification has performed well on both classes.* ***Such arguments are founded on unrealistic conditions (e.g. on the use of a gold standard reference). In reality, the magnitude of the MCC is influenced greatly by class imbalance and reference data imperfections.”***

*The text highlighted in bold above aims to address the point flagged by the referee.*

Line 690 should be changed from "all four of them area high” to "all four of them are high"

*The change has been made.*

Finally, this article misses specific recommendations to address the shortcomings of classification metrics when the reference data has errors. The article mentions many times that imperfect reference data will adversely affect the classification metrics, but in that case even the confusion matrices will be unreliable. The author should share some insights on how to address this situation, since the article is aimed at the practitioner.

*This is a good point but discussion on the methods to address the problems would be a whole new paper it itself. However, I agree with the referee that it would be useful to point to means to address the problems. The very final sentence of the concluding section has been changed and points the interested reader to 3 articles that discuss several approaches that may be used – specifically to references 20, 63 and 71 (the latter is a new reference added as a result of the referee’s comments).*

*I am grateful to the referees and editorial team for their comments on the article.*
